# The Role of Radiology in Progressive Fibrosing Interstitial Lung Disease

**DOI:** 10.3389/fmed.2021.679051

**Published:** 2022-01-13

**Authors:** Ahmad Abu Qubo, K. M. Capaccione, Elana J. Bernstein, Maria Padilla, Mary Salvatore

**Affiliations:** ^1^Department of Radiology, Columbia University Irving Medical Center, New York, NY, United States; ^2^Department of Rheumatology, Columbia University Irving Medical Center, New York, NY, United States; ^3^Department of Pulmonary Medicine, Mount Sinai Medical Center, New York, NY, United States

**Keywords:** progressive fibrosing interstitial lung disease, usual interstitial pneumonitis, nonspecific interstitial pneumonitis, hypersensitivity pneumonitis, sarcoidosis

## Abstract

In this article, we describe the role of radiology for diagnosis and follow-up of progressive fibrosing interstitial lung disease (PF-ILD). Patients with PF-ILD are at increased risk for early death without treatment. Clinical diagnosis of PF-ILD has been described in the literature. This manuscript reviews the radiographic diagnosis of PF-ILD and the unique CT characteristics associated with specific types of fibrosis. Ultimately, we believe that radiology has the potential to recognize progression early and thus make an important contribution to the multidisciplinary discussion for this important diagnosis.

## Introduction

In broad terms, there are two types of interstitial lung diseases (ILDs); one of which is primarily inflammatory with the potential for complete resolution but with the possibility of transformation into a fibrotic phenotype, the other is a primarily fibrotic ILD and largely irreversible (1). The treatment of an inflammatory ILD is different from its fibrotic counterpart thus correct diagnosis has important implications. Progressive fibrosing interstitial lung disease (PF-ILD) is defined as a subtype of interstitial lung disease which advances despite current state of the art treatment and predicts a poor outcome (2). Inflammatory ILDs can transform to the progressive fibrotic type, which may then benefit from anti-fibrotic treatment strategies to slow the decline of forced vital capacity (FVC) (3).

Nintedanib is approved for treatment of idiopathic pulmonary fibrosis (IPF), and has demonstrated improved outcomes with diminished FVC decline compared to placebo in PF- ILD patients (3). The RELIEF study, a phase 2b trial, was terminated prematurely due to slow enrollment but found that adding pirfenidone to treatment regimen in patients with non-IPF slowed disease progression (4). Raghu warns that the use of a single agent to treat all progressive fibrotic lung diseases should not deter the clinician from evaluating the underlying cause as there may be specific interventions that could lead to improved patient outcomes (4). Defining this phenotype has implications that go beyond therapy with the opportunity for early referral to lung transplantation and/or the initiation of advanced care planning (5).

There is limited experience with the prevalence and incidence of the progressive phenotype of ILD (6). One source, which excluded IPF, estimates the progressive phenotype to be 13% to 40% depending on the underlying diagnosis (7). The diagnosis of progressive disease relies upon a decline in pulmonary function tests, progressive shortness of breath, and more extensive disease on imaging (2). In the INBUILD trial, which studied the effect of Nintedanib on the progressive phenotype, the criteria for progression were: 10% decrease of the predicted FVC, 5 to 10% decrease in predicted FVC with progressive dyspnea or increased radiographic fibrosis, or progressive dyspnea with increased radiographic fibrosis, all within 24 months prior to screening and despite conventional therapy (3). Regardless of how this term is defined, it is best established through a multidisciplinary discussion. Radiology plays a vital role in the diagnosis of the progressive phenotype by identifying increased pulmonary fibrosis. Collins et al. argue that the pattern and extent of fibrosis could affect clinical outcomes more than the diagnosis (8). The INBUILD criteria for progression were used in a retrospective study in the UK with 1,749 patients with ILD other than IPF. Two hundred and fifty three patients (14.5%) met the criteria for a progressive phenotype. This subset of patients had worse mortality compared to the non-progressive ILD and similar mortality to IPF (9). Other research papers have found similar results (10). The intent of our paper is to describe imaging features of the progressive phenotype of ILD. Ultimately a multidisciplinary discussion is necessary for the accurate diagnosis.

## Usual Interstitial Pneumonitis (UIP)

The 2018 American thoracic society IPF guidelines provide four levels of confidence for UIP diagnosis. These include: UIP, probable UIP, indeterminate for UIP and a pattern suggesting alternative diagnosis. UIP is defined with sub pleural basilar predominant fibrosis, honeycombing, and the absence of features that would suggest another diagnosis. If there is no honeycombing, the pattern is a “probable UIP” pattern which previously was termed “possible UIP” (11).

UIP pattern on CT is often associated with the clinical diagnosis of idiopathic pulmonary fibrosis (IPF) that has a life expectancy of 3 to 4 years without treatment (12). Patients with a UIP pattern are more likely to be older in age, male gender, and smokers (13). This pattern carries a worse prognosis and an increased risk of death regardless of the underlying cause (14). More frequent acute exacerbations are also seen with a UIP pattern (15). It is worth mentioning that a UIP pattern does not automatically equal a diagnosis of IPF as other interstitial lung diseases can have this pattern on HRCT (16). Drug toxicity, collagen vascular disease and chronic hypersensitivity pneumonitis and familial pulmonary fibrosis can also present with this pattern (17, 18). Several signs may help differentiate UIP pattern associated with IPF from a UIP pattern of other etiologies, these include the straight-edge sign, anterior upper lobe sign, and exuberant honeycombing, which are more likely to denote an underlying connective tissue disease rather than IPF (19). Non-IPF ILD's, which usually have better survival than IPF, carry similar mortality to IPF if radiologic honeycombing is present (20).

Fibrosis of the lung increases the density of the lung which is measured by Hounsfield units (HU). On chest CT, the normal lung measures from −850HU. In contrast, fibrotic lung appears whiter on CT with HUs of approximately −500 HU. Analysis of extent of high density areas allows the radiologist to provide a visual quantification of the amount of fibrosis (21). A grading system can provide a visual quantification of fibrosis (22). Follow up imaging can be used to assess the extent of conversion of normal lung to fibrotic lung. Therefore, it follows that a person who has progressive fibrosis would have a rate of change greater than zero and there is the opportunity to differentiate rapid progressors from slow progressors.

**Figure 1:**
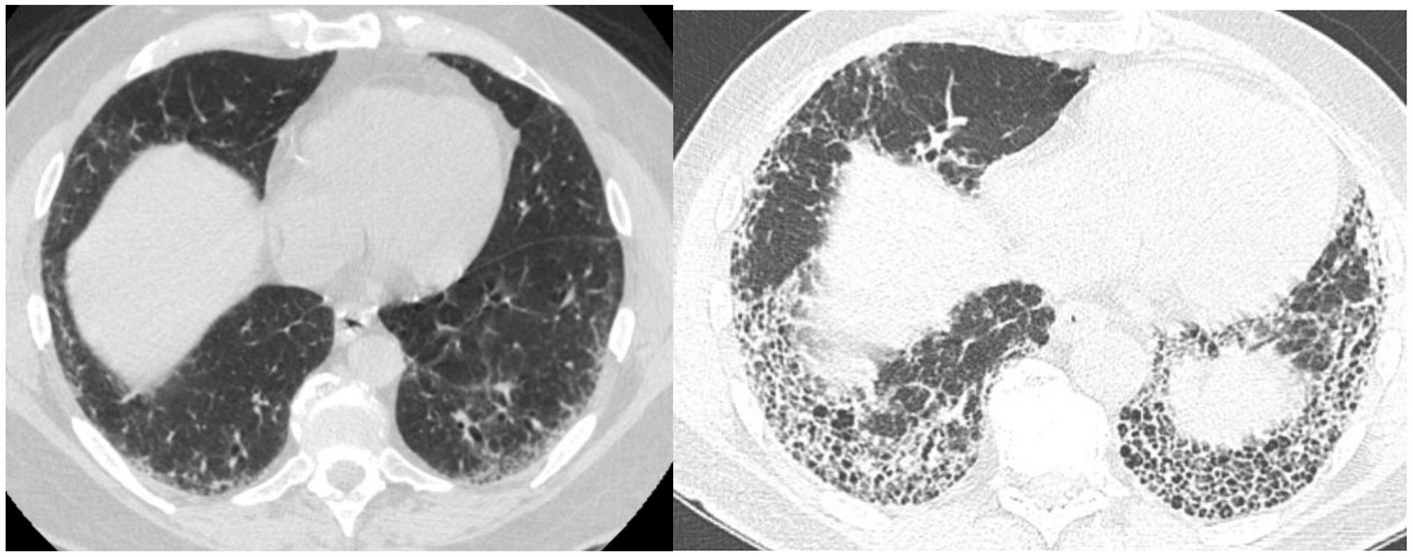
Change from *probable UIP* pattern to *UIP* pattern indicative of progressive fibrosis.

Not all patients who progress with IPF increase their extent of disease. Some progress by changing from a probable UIP to a UIP pattern with the development of honeycombing (Figure 1). Previously we reviewed 103 patients with a working diagnosis of IPF, 68 had a probable UIP pattern on the initial CT, 47% progressed to a UIP pattern with honeycombing (HC) over 51 months, therefore having a progressive phenotype. Thirty five patients had HC on the initial CT scan of which 57% increased the extent of disease over 31 months, also progressive disease. Enlarged pulmonary artery and emphysema were associated with disease progression (22). Serial imaging is used to evaluate progression which is not necessarily discovered between the first and second HRCT scans. Serial CT scans have the added benefit of the early identification of lung cancer (23).

**Figure 2:**
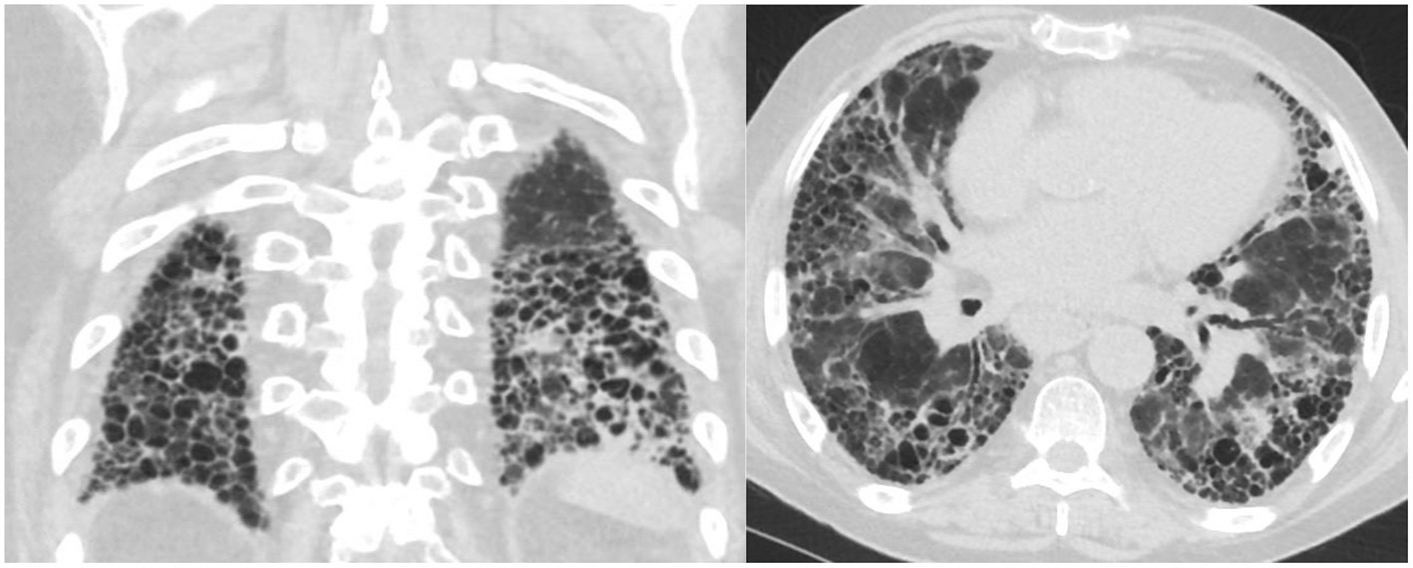
Axial and coronal images with straight edge sign characteristic of a UIP pattern associated with CTD.

Not all patients with connective tissue disease have an NSIP pattern, some patients have a UIP pattern with exuberant honeycombing and the straight edge sign (Figure 2) which can also progress in extent over time (24).

## Nonspecific Interstitial Pneumonitis (NSIP)

**Figure 3:**
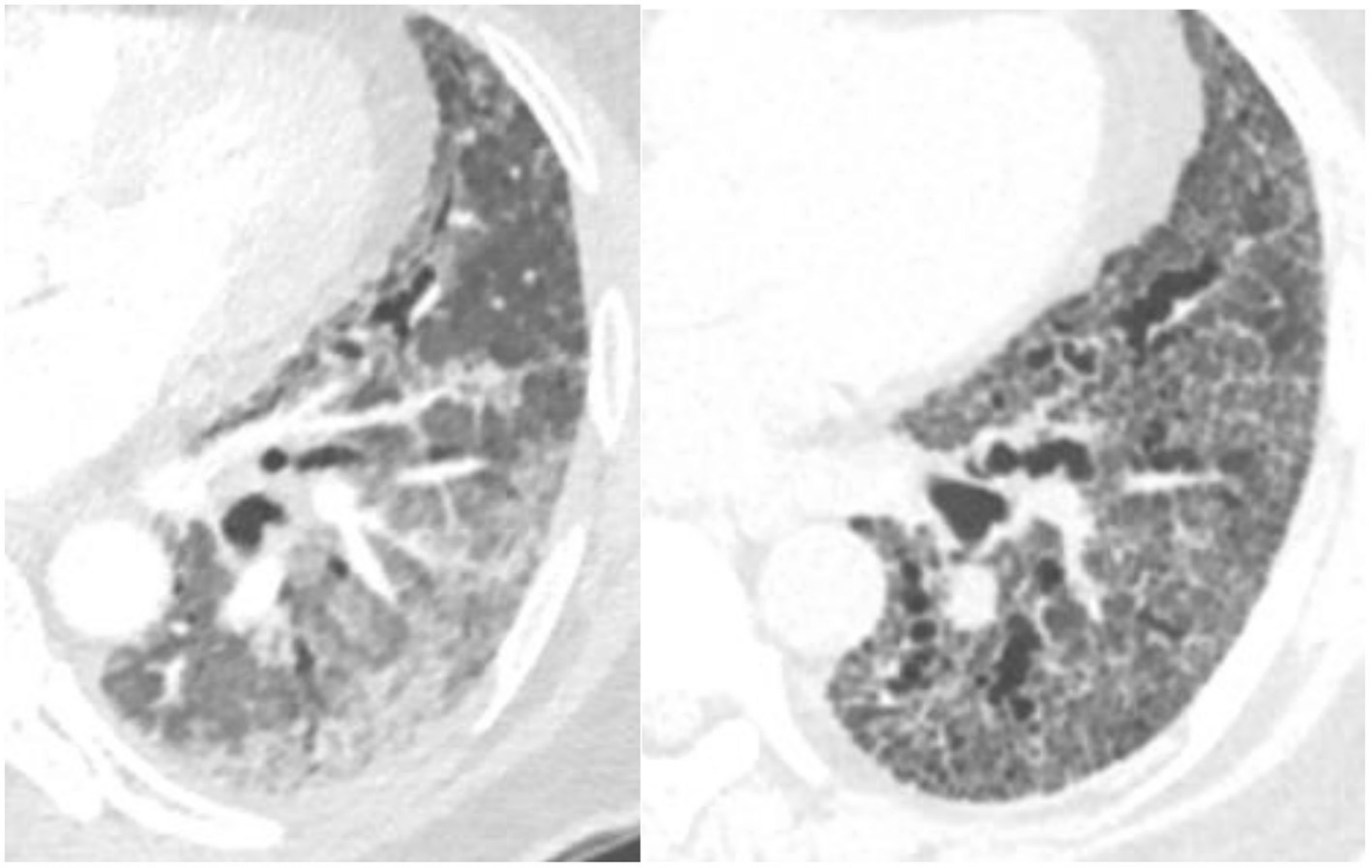
Evolution of scleroderma from a cellular NSIP pattern to a mixed cellular and fibrotic pattern with bronchiectasis over the course of 10 months consistent with progressive disease.

NSIP is a homogeneous, lower lung predominant type of fibrosis typically associated with connective tissue disease (CTD) (25). People with an NSIP pattern are more likely to be younger, and female gender (13). It has an overall better prognosis with fewer exacerbations compared to UIP (26), the median survival is >9 years (13). The extent of disease can be measured visually and progression noted when the extent of normal lung decreases. The radiologists can also describe the progression from non-fibrotic cellular NSIP with its predominant ground-glass opacity to mixed cellular and fibrotic NSIP with ground glass opacities and bronchiectasis and eventually fibrotic NSIP with the resolution of the majority of ground glass and addition of honeycombing in some patients (Figure 3). In a study of 108 patients with a working diagnosis of CTD, the radiographic pattern of disease changed over time in 35%. Sixty-three percent of patients had increased extent of disease on CT over time (26). The progression from a cellular phenotype to fibrosis is an important radiographic observation and along with patients clinical status can define a progressive phenotype.

**Figure 4:**
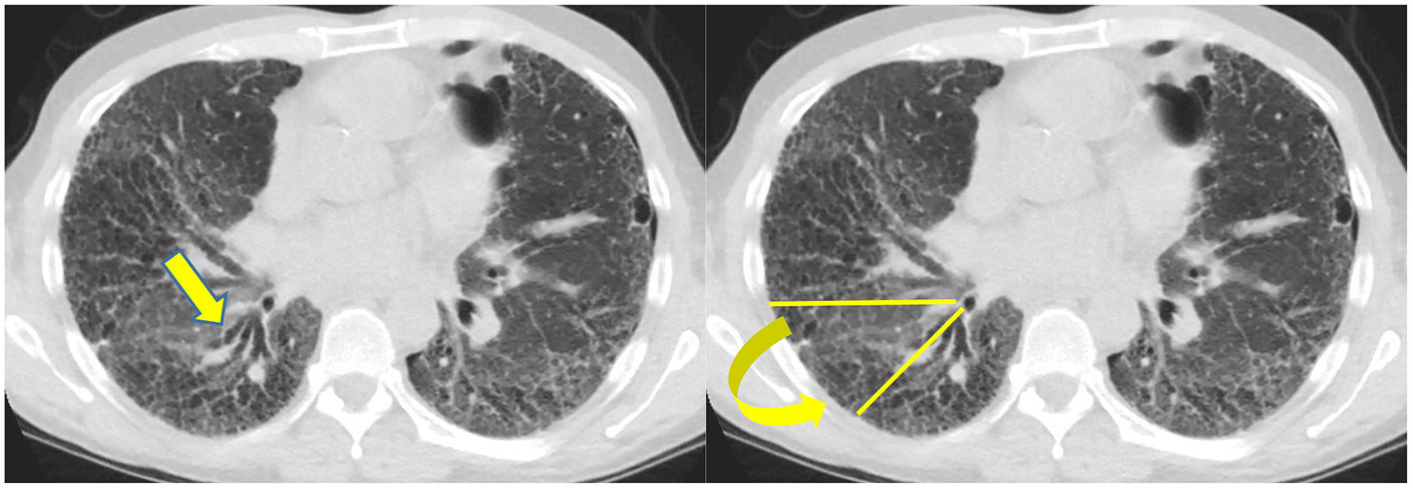
Typically the right lower lobe anterior bronchus (yellow arrow) follows a horizontal course but in this patient it is displaced posteriorly by volume loss and fibrosis. The displacement of the bronchus can be followed to document disease progression.

Biomarkers are medical signs which can be measured and used to diagnose disease (27). Radiology can function as a biomarker. In fibrotic NSIP, the lower lobe predominant scarring diminishes the volume of the inferior lung and displaces the major fissures posteriorly, more so than UIP, which is also a lower lobe predominant fibrotic disease. An increase in the amount of volume loss for this disease can be used in part to identify a progressive phenotype (28) (Figure 4).

## Hypersensitivity Pneumonitis (HP)

**Figure 5:**
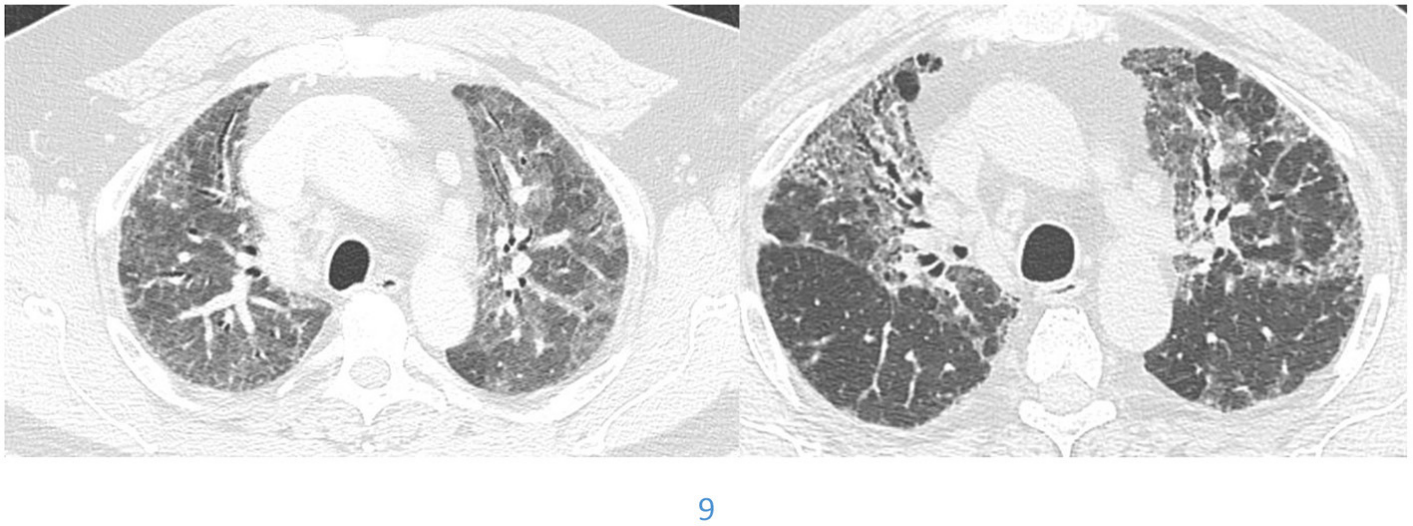
Evolution of hypersensitivity pneumonitis from non-fibrotic to fibrotic phenotype over a 39 month period.

Patients with prolonged inhaled antigen exposure are at risk for permanent scarring of the lung. Patients with this pattern are more likely to be females and non-smokers (29). The pathology demonstrates fibrosis along the airways which may be associated with poorly formed granulomas (29). The latest guidelines for HP diagnosis were set up by the ATS in 2020. Three categories of confidence for HP diagnosis were listed, typical HP, compatible with HP and indeterminate for HP (30). Typical HP shows features of lung infiltration, with either mosaic attenuation or ground glass opacifications, plus features of small airway disease, with either air trapping or indistinct centrilobular nodules. Both of these criteria must be diffuse in distribution. The guidelines simplified the radiographic diagnosis further into two subtypes, fibrotic and non-fibrotic. The fibrotic phenotype is associated with greater traction bronchiectasis, architectural distortion and volume loss. Just like NSIP and UIP, the extent of disease can increase and suggest progression. In addition, the stage of disease can evolve from mostly non-fibrotic ground glass opacities which may resolve with antigen removal to a fibrotic phase with predominantly bronchiectasis (Figure 5). The change in stage with increased fibrosis represent disease progression (31).

## Sarcoidosis

**Figure 6:**
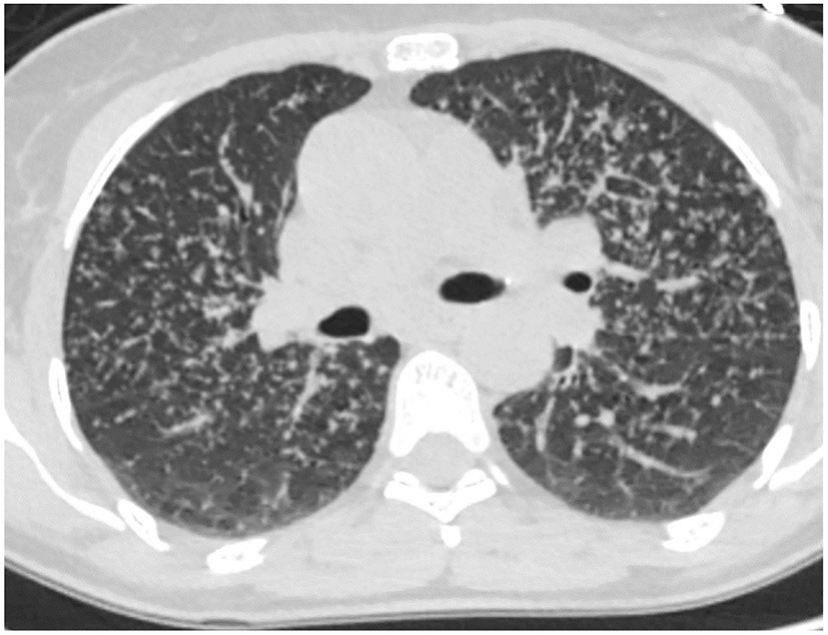
Non-fibrotic sarcoidosis with pulmonary nodules in a peri-lymphatic distribution.

**Figure 7:**
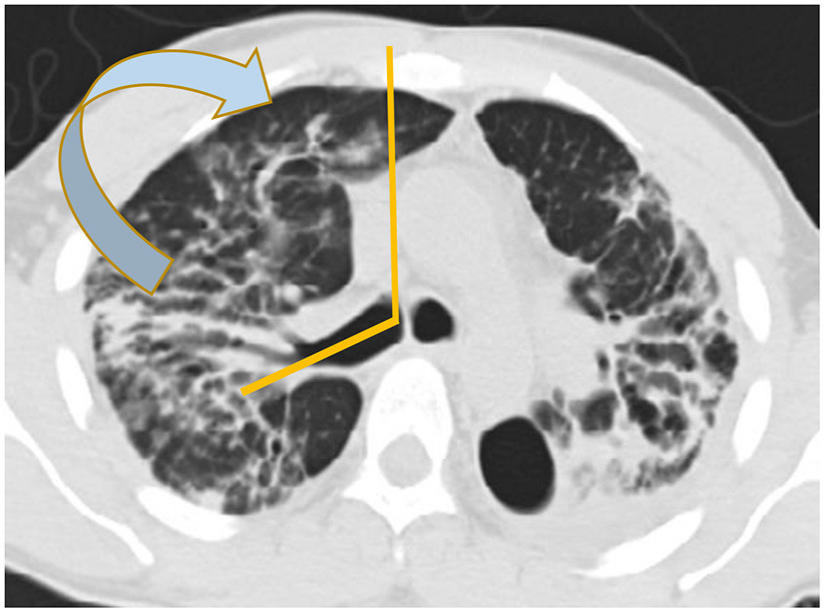
Stage 4 sarcoidosis is associated with progressive loss of volume of the upper lobes with displacement of the right upper lobe bronchus posteriorly as seen in this image.

There are basically two main types of sarcoidosis, fibrotic and non-fibrotic (32). The non-fibrotic type of sarcoid has pulmonary nodules and lymphadenopathy (Figure 6). The major feature of the progression of the fibrotic type of disease is volume loss with tenting of the hemi-diaphragms. Fibrotic sarcoid primarily affects the posterior aspect of the upper lobes and as such displaces the right upper lobe bronchus (32). In particular, the radiologist can measure the angle between a line traversing the right upper lobe bronchus and a sagittal line connecting the sternum to the vertebral body. The angle is called the Right Upper Lobe Bronchus Angle (RUL-BA) (Figure 7). The angle increases with the fibrotic phase of the disease and can be used as a biomarker for disease progression (32).

## Conclusion

Progressive fibrosing interstitial lung disease occurs in a subset of individuals whose interstitial lung disease progresses despite appropriate treatment. Radiology contributes to the accurate diagnosis of the progressive phenotype by recognizing increasing fibrosis over time (Table 1).

**Table 1:** Radiology features of progressive fibrotic interstitial lung disease.

	**General CT signs of** **progression**	**Specific CT signs of** **progression**
UIP	Increase extent of fibrosis	Probable UIP to UIP pattern
NSIP	Increase extent of fibrosis	Cellular to fibrotic pattern
CHP	Increase extent of fibrosis	Cellular to fibrotic pattern
Sarcoid	Increase extent of fibrosis	Nodular to fibrotic pattern

## Author Contributions

All authors listed have made a substantial, direct, and intellectual contribution to the work and approved it for publication.

## Conflict of Interest

MS: speaker, advisory board, and grant funding from Genentech and Boehringer Ingelheim. MP: speaker for Vindico Medical education and France Foundation and a consultant for Genentech and Boehringer Ingelheim and Paradigm Medical Communications and has received a research grant from Boehringer Ingelheim. The remaining authors declare that the research was conducted in the absence of any commercial or financial relationships that could be construed as a potential conflict of interest.

## Publisher's Note

All claims expressed in this article are solely those of the authors and do not necessarily represent those of their affiliated organizations, or those of the publisher, the editors and the reviewers. Any product that may be evaluated in this article, or claim that may be made by its manufacturer, is not guaranteed or endorsed by the publisher.

## References

[1] Kolb M Vašáková M. The natural history of progressive fibrosing interstitial lung diseases. Respir Res. (2019) 20:57. 10.1186/s12931-019-1022-1 30871560 PMC6417262

[2] George PM Spagnolo P Kreuter M Altinisik G Bonifazi M Martinez FJ. Progressive fibrosing interstitial lung disease: clinical uncertainties, consensus recommendations, research priorities. Lancet Respir Med. (2020) 8:925–34. 10.1016/S2213-2600(20)30355-6 32890499

[3] Flaherty KR Wells AU Cottin V Devaraj A Walsh S Inoue Y. Nintedanib in progressive fibrosing interstitial lung diseases. N Engl J Med. (2019) 381:1718–27. 10.1056/NEJMoa1908681 31566307

[4] Raghu G. Nintedanib in progressive fibrosing interstitial lung diseases. N Engl J Med. (2020) 382:779–80. 10.1056/NEJMc1917224 32074430

[5] De Sadeleer LJ Goos T Yserbyt J Wuyts WA. Towards the essence of progressiveness: bringing progressive fibrosing interstitial lung disease (PF-ILD) to the next stage. J Clin Med. (2020) 9:1722. 10.3390/jcm9061722 32503224 PMC7355916

[6] Cottin V. Treatment of progressive fibrosing interstitial lung diseases: a milestone in the management of interstitial lung diseases. Eur Respir Rev. (2019) 28:190109. 10.1183/16000617.0109-2019 31578213 PMC9488849

[7] Olson A Hartmann N Patnaik P Wallace L Schlenker-Herceg R Nasser M. Estimation of the prevalence of progressive fibrosing interstitial lung diseases: systematic literature review and data from a physician survey. Adv Ther. (2021) 38:854–67. 10.1007/s12325-020-01578-6 33315170 PMC7889674

[8] Collins BF Raghu G. Antifibrotic therapy for fibrotic lung disease beyond idiopathic pulmonary fibrosis. Eur Respir Rev. (2019) 28:190022. 10.1183/16000617.0022-2019 31578210 PMC9489066

[9] Simpson T Barratt SL Beirne P Chaudhuri N Crawshaw A Crowley LE. The burden of progressive fibrotic interstitial lung disease across the UK. Eur Respir J. (2021) 58:2100221. 10.1183/13993003.00221-2021 33678609 PMC8264777

[10] Komatsu M Yamamoto H Kitaguchi Y Kawakami S Matsushita M Uehara T. Clinical characteristics of non-idiopathic pulmonary fibrosis, progressive fibrosing interstitial lung diseases: A single-center retrospective study. Medicine. (2021) 100:e25322. 10.1097/MD.0000000000025322 33787626 PMC8021292

[11] Raghu G Remy-Jardin M Myers JL Richeldi L Ryerson CJ Lederer DJ. Diagnosis of idiopathic pulmonary fibrosis. An official ATS/ERS/JRS/ALAT clinical practice guideline. Am J Respir Crit Care Med. (2018) 198:e44–68. 30168753 10.1164/rccm.201807-1255ST

[12] Raghu G Chen SY Yeh WS Maroni B Li Q Lee YC. Idiopathic pulmonary fibrosis in US Medicare beneficiaries aged 65 years and older: incidence, prevalence, and survival, 2001-11. Lancet Respir Med. (2014) 2:566–72. 10.1016/S2213-2600(14)70101-8 24875841

[13] Ebner L Christodoulidis S Stathopoulou T Geiser T Stalder O Limacher A. Meta-analysis of the radiological and clinical features of usual interstitial pneumonia (UIP) and nonspecific interstitial pneumonia (NSIP). PLoS ONE. (2020) 15:e0226084. 10.1371/journal.pone.0226084 31929532 PMC6957301

[14] Wong AW Ryerson CJ Guler SA. Progression of fibrosing interstitial lung disease. Respir Res. (2020) 21:32. 10.1186/s12931-020-1296-3 31996266 PMC6988233

[15] Kolb M Bondue B Pesci A Miyazaki Y Song JW Bhatt NY. Acute exacerbations of progressive-fibrosing interstitial lung diseases. Eur Respir Rev. (2018) 27:180071. 10.1183/16000617.0071-2018 30578331 PMC9488799

[16] Wuyts WA Cavazza A Rossi G Bonella F Sverzellati N Spagnolo P. Differential diagnosis of usual interstitial pneumonia: when is it truly idiopathic? Eur Respir Rev. (2014) 23:308–19. 10.1183/09059180.00004914 25176967 PMC9487316

[17] Aburto M Herráez I Iturbe D Jiménez-Romero A. Diagnosis of idiopathic pulmonary fibrosis: differential diagnosis. Med Sci (Basel). (2018) 6:73. 10.3390/medsci6030073 30181506 PMC6164303

[18] Bennett D Mazzei MA Squitieri NC Bargagli E Refini RM Fossi A. Familial pulmonary fibrosis: Clinical and radiological characteristics and progression analysis in different high resolution-CT patterns. Respir Med. (2017) 126:75–83. 10.1016/j.rmed.2017.03.020 28427553

[19] Chung JH Landeras L. Probable UIP: What is the evidence that compels this classification and how is it different from the indeterminate category? Semin Roentgenol. (2019) 54:15–20. 10.1053/j.ro.2018.12.006 30684992

[20] Adegunsoye A Oldham JM Bellam SK Montner S Churpek MM Noth I. Computed tomography honeycombing identifies a progressive fibrotic phenotype with increased mortality across diverse interstitial lung diseases. Ann Am Thorac Soc. (2019) 16:580–8. 10.1513/AnnalsATS.201807-443OC 30653927 PMC6491052

[21] Salvatore M Henschke CI Yip R Jacobi A Eber C Padilla M. JOURNAL CLUB: Evidence of interstitial lung disease on low-dose chest ct images: prevalence, patterns, and progression. AJR Am J Roentgenol. (2016) 206:487–94. 10.2214/AJR.15.15537 26700157

[22] Salvatore M Singh A Yip R Fevrier E Henschke CI Yankelevitz D. Progression of probable UIP and UIP on HRCT. Clin Imaging. (2019) 58:140–4. 10.1016/j.clinimag.2019.07.003 31326632

[23] Walsh S Devaraj A Enghelmayer JI Kishi K Silva RS Patel N. Role of imaging in progressive-fibrosing interstitial lung diseases. Eur Respir Rev. (2018) 27:180073. 10.1183/16000617.0073-2018 30578332 PMC9488692

[24] Chung JH Cox CW Montner SM Adegunsoye A Oldham JM Husain AN. CT features of the usual interstitial pneumonia pattern: differentiating connective tissue disease-associated interstitial lung disease from idiopathic pulmonary fibrosis. AJR Am J Roentgenol. (2018) 210:307–13. 10.2214/AJR.17.18384 29140119 PMC12525794

[25] Salvatore M Smith ML. Cross sectional imaging of pulmonary fibrosis translating pathology into radiology. Clin Imaging. (2018) 51:332–6. 10.1016/j.clinimag.2018.06.013 29960266

[26] Salvatore M Mathur M Fang Guo L O'Connor T Padilla M. Connective tissue disease and its evolution on chest CT. Curr Trends Clin Med Imaging. (2019) 3:555621. 10.19080/CTCMI.2019.03.555621

[27] Strimbu K Tavel JA. What are biomarkers? Curr Opin HIV AIDS. (2010) 5:463–6. 10.1097/COH.0b013e32833ed177 20978388 PMC3078627

[28] Robbie H Wells AU Jacob J Walsh S Nair A Srikanthan A. Visual and automated CT measurements of lung volume loss in idiopathic pulmonary fibrosis. AJR Am J Roentgenol. (2019) 213:318–24. 10.2214/AJR.18.20884 31063425

[29] Magee AL Montner SM Husain A Adegunsoye A Vij R Chung JH. Imaging of hypersensitivity pneumonitis. Radiol Clin North Am. (2016) 54:1033–46. 10.1016/j.rcl.2016.05.013 27719974 PMC6571018

[30] Raghu G Remy-Jardin M Ryerson CJ Myers JL Kreuter M Vasakova M. Diagnosis of hypersensitivity pneumonitis in adults. An official ATS/JRS/ALAT clinical practice guideline. Am J Respir Crit Care Med. (2020) 202:e36–69. 10.1164/rccm.202005-2032ST 32706311 PMC7397797

[31] Salisbury ML Gu T Murray S Gross BH Chughtai A Sayyouh M. Hypersensitivity pneumonitis: radiologic phenotypes are associated with distinct survival time and pulmonary function trajectory. Chest. (2019) 155:699–711. 10.1016/j.chest.2018.08.1076 30243979 PMC6514431

[32] Salvatore M Toussie D Pavlishyn N Yankelevitz D O'Connor T Henschke C. The right upper lobe bronchus angle: A tool for differentiating fibrotic and non-fibrotic sarcoidosis. Sarcoidosis Vasc Diffuse Lung Dis. (2020) 37:99–103. 10.36141/svdld.v37i2.8965 33093775 PMC7569551

